# Improved Detection Accuracy of Chronic Vertebral Compression Fractures by Integrating Height Loss Ratio and Deep Learning Approaches

**DOI:** 10.3390/diagnostics14222477

**Published:** 2024-11-06

**Authors:** Jemyoung Lee, Heejun Park, Zepa Yang, Ok Hee Woo, Woo Young Kang, Jong Hyo Kim

**Affiliations:** 1 Department of Applied Bioengineering, Graduate School of Convergence Science and Technology, Seoul National University, Seoul 08826, Republic of Korea; jaymlee0407@snu.ac.kr; 2 ClariPi Research, ClariPi Inc., Seoul 03088, Republic of Korea; 3 Department of Radiology, Korea University Guro Hospital, Seoul 08308, Republic of Korea; eirbadmin@kumc.or.kr (H.P.); yangzepa@gmail.com (Z.Y.); wokhee@korea.ac.kr (O.H.W.); 4 Department of Radiology, Seoul National University College of Medicine, Seoul 03080, Republic of Korea; 5 Department of Radiology, Seoul National University Hospital, Seoul 03080, Republic of Korea; 6 Center for Medical-IT Convergence Technology Research, Advanced Institutes of Convergence Technology, Suwon 16229, Republic of Korea

**Keywords:** vertebral compression fracture, height loss ratio, deep learning, spine, computed tomography

## Abstract

Objectives: This study aims to assess the limitations of the height loss ratio (HLR) method and introduce a new approach that integrates a deep learning (DL) model to enhance vertebral compression fracture (VCF) detection performance. Methods: We conducted a retrospective study on 589 patients with chronic VCFs. We compared four different methods: HLR-only, DL-only, a combination of HLR and DL for positive VCF, and a combination of HLR and DL for negative VCF. The models were evaluated using dice similarity coefficient, sensitivity, specificity, and area under the receiver operating characteristic curve (AUROC). Results: The combined method (HLR + DL, positive) demonstrated the best performance with an AUROC of 0.968, sensitivity (94.95%), and specificity (90.59%). The HLR-only and the HLR + DL (negative) also showed strong discriminatory power, with AUROCs of 0.948 and 0.947, respectively. The DL-only model achieved the highest specificity (95.92%) but exhibited lower sensitivity (82.83%). Conclusions: Our study highlights the limitations of the HLR method in detecting chronic VCFs and demonstrates the improved performance of combining HLR with DL models.

## 1. Introduction

The vertebral compression fracture (VCF) is often caused by low bone density in elderly patients. VCFs are most common in postmenopausal populations, which have lower bone density than before. The ‘Osteoporosis and Osteopenia Fractures Fact Sheet 2023’ by the Korean Society for Bone and Mineral Research reported that 50.6% of osteoporotic fractures in female patients were diagnosed with VCF, and in male patients in their 70s, 50.1% of fractures were found to be VCF. In female patients over 50 years old, almost 330,000 patients were diagnosed with osteoporotic fractures in 2022. Additionally, the elderly male group was not exempt from fractures, as more than 100,000 cases of osteoporuotic fractures were diagnosed in 2022 alone in male patients over 50 years old. The mortality rate within one year after a vertebral fracture was 6%, with men having a higher mortality rate than women after a fracture. Similarly, in the United States, there were approximately 1.5 million VCF patients annually [1]. We are currently in a super-aging era, with the older population increasing year by year. As a result, the number of VCF patients is rising every year.

For the diagnosis of VCF, plain radiographs are clinically the initial investigation, and if some disorder is detected, CT or MRI are recommended for further specific examination. Numerous studies have been conducted to develop deep learning (DL) models based on radiographs using classification, detection, and segmentation techniques [2,3,4,5]. However, on radiograph images, the 2D lateral projection cannot accurately depict the exact features of VCF in each vertebra. The ribs overlapping the thoracic, thoracolumbar junction vertebrae, and upper lumbar levels (L1–L2), as well as internal organs like the liver and intestines, can obstruct an accurate diagnosis of VCF. For this reason, CT or MRI is considered the reference standard for VCF diagnosis [6,7]. Although MRI can detect edema or soft tissue degenerative lesions related to VCF, the longer acquisition time of MRI is a critical issue, which often leads to the preference for CT in diagnosing VCF. VCF can be identified in sagittal-reformatted CT images, where cortical bone deformation and trabecular bone HU variations are apparent.

For the quantitative measurements of VCF, the majority of research studies used the Genant classification method [8]. We measured the height of the vertebra at three points: anterior, middle, and posterior. The posterior portion, located near the pedicle and spinal process, serves as the standard height for each vertebra. The anterior and middle heights of the vertebra were then compared to the posterior height. We calculated the height loss ratio (HLR) for each vertebra, and the VCF was classified into four different groups: normal (HLR < 20%), mild (20% ≤ HLR < 25%), moderate (25% ≤ HLR < 40%), and severe (HLR ≥ 40%). However, the Genant classification requires drawing three vertical lines on each individual vertebra, which is time-consuming. Detecting fractures in bone images is a labor-intensive task that demands careful manual examination by a highly skilled radiologist [9]. Additionally, we did not classify all vertebrae based on the Genant classification clinically. While HLR-based Genant classification can serve as a reference for diagnosing VCF, it is not sufficient as a standalone criterion. We also need to assess the degree of deformity of vertebral contour on CT images, which is challenging to quantify for every vertebra [7].

In this study, we aimed to figure out the limitations of HLR method for identifying clinically classified VCF vertebrae. Additionally, we proposed a new approach for detecting VCF features using a vertebral contour-based detection model, and we demonstrated the feasibility of combining algorithms—specifically, the quantitative method of HLR and the qualitative method of DL—to improve the detection performance for chronic VCF.

## 2. Materials and Methods

### 2.1. Patients Datasets

**Figure 1:**
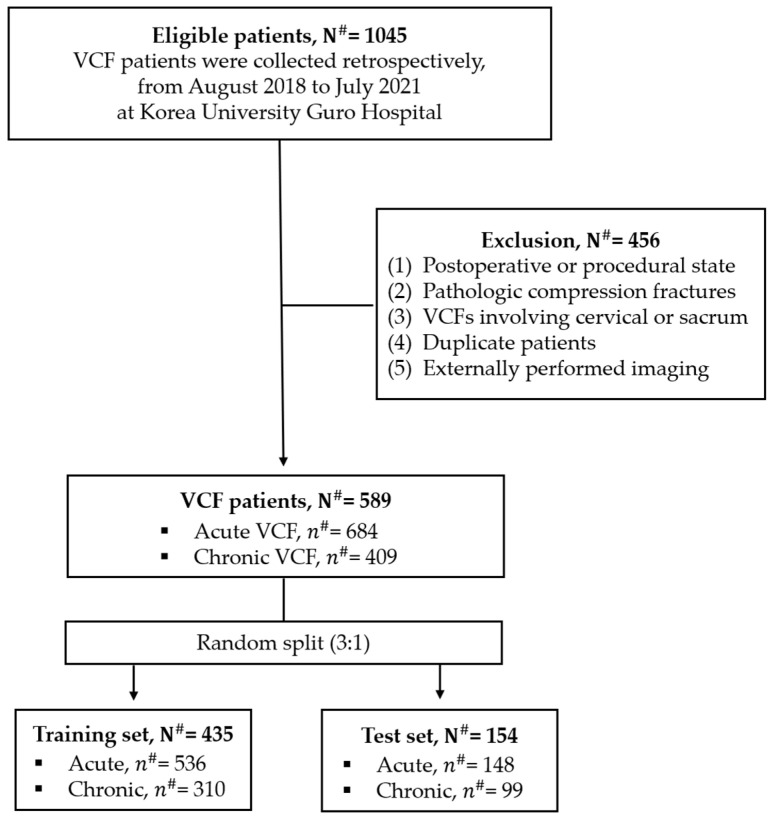
Flowchart of participants. N^#^ means the number of patients and *n*^#^ means the number of vertebral bodies.

This retrospective study complied with the principles of the Helsinki Declaration and was approved by the Institutional Review Board (IRB) of Korea University Guro Hospital. The study cohort for benign VCFs was identified by querying the institutional PACS database for thoracic or lumbar spine CT examinations performed between August 2018 and July 2021. The search criteria included report texts containing the terms “compression fracture”, “burst fracture”, or “compression deformity”. The initial cohort comprised 1045 patients. Exclusion criteria were applied as follows: (1) postoperative or procedural state following any form of spinal surgery, including the insertion of metallic hardware or bone cement, (2) pathologic compression fractures related to tumors or infections, (3) compression fractures involving the cervical spine or sacrum, (4) duplicate imaging in the same patient, and (5) imaging performed externally. Two radiologists, each with 20 years of experience in musculoskeletal imaging, along with a third-year radiology trainee, reviewed the cases and classified the benign VCFs as either acute or chronic. Benign VCFs were defined as traumatic or osteoporotic fractures not associated with malignancy. Acute VCFs were characterized by the presence of cortical disruption, low-attenuation fissures, or sclerotic lines on CT imaging, while chronic VCFs were identified as compressive deformities lacking these acute features. A total of 589 patients met the inclusion criteria, and we only considered chronic VCFs in our study (Figure 1). As this study conducted an in-depth analysis on a vertebral body level, we examined how many vertebrae had chronic fractures and how many were normal. The training dataset included 310 chronic fracture cases, and the test dataset comprised 99 chronic fractures and 882 normal vertebrae, resulting in a total of 981 vertebrae used for performance evaluation.

The radiologists labeled all VCFs on sagittal-reformatted CT images by creating bounding box masks around the affected vertebrae. All CT images were scanned using nine different types of reconstruction kernels with a 2 mm slice thickness and without an interval gap across five different CT manufacturer models (SOMATOM Force, SOMATOM Definition AS+, SOMATOM Definition Edge, Siemens Healthineers, Erlangen, Germany; Brilliance 64, Philips Healthcare, Best, The Netherlands; and Aquilion ONE, Canon Medical Systems, Otawara, Japan). All selected images were saved in bone settings (window width 1500 and window level 300) from the picture archiving and communication system (PACS).

### 2.2. Deep Learning Model Development

#### 2.2.1. Study Settings

We developed two deep learning models: one was a spine segmentation model using the U-Net Transformer (UNETR) architecture, and the other was a VCF detection model using the YOLOv9 architecture. For the training and validation of our DL models, we used two different datasets. For the spine segmentation model, we used 324 cases (31,971 slices) from the VerSe2020 open dataset, which provided specific vertebral masks labeled for each spinal level [10,11,12]. The CT scans in this dataset were obtained from two different manufacturers and utilized five distinct CT models: Philips Brilliance 64, iCT, IQon, and Siemens SOMATOM Definition AS and AS+. The VerSe2020 dataset was collected with all scans at 120 kVp, featuring slice thicknesses between 0.9 mm and 2.0 mm, and pixel spacing ranging from 0.81 × 0.81 mm to 0.88 × 0.88 mm. Among the 324 cases, 25.6% (83 cases) were categorized as normal, 17.6% (57 cases) as osteopenia, and 56.8% (184 cases) as osteoporosis. The data were randomly divided into training and test sets, with 243 cases allocated for training and 81 cases for testing, following a 3:1 ratio. As shown in Figure 1, we randomly split the data into training and test sets at a 3:1 ratio, using 435 patient CT datasets for training the VCF detection model with the generation of spine contour masks using the spine segmentation model, and tested with 154 cases.

#### 2.2.2. Spine Segmentation

We used the UNETR architecture for spine segmentation. The UNETR model integrates the Vision Transformer (ViT) architecture with the traditional U-Net framework to improve performance in medical image segmentation tasks [13,14]. U-Net, which is primarily based on a convolutional neural network (CNN), segments images by reconstructing high-resolution features using an encoder–decoder architecture [15]. Transformers, originally developed for natural language processing (NLP), utilize a self-attention mechanism that effectively captures long-range dependencies [16]. The UNETR design enables various combinations of encoders and decoders, making it versatile for different datasets and challenges.

**Figure 2:**
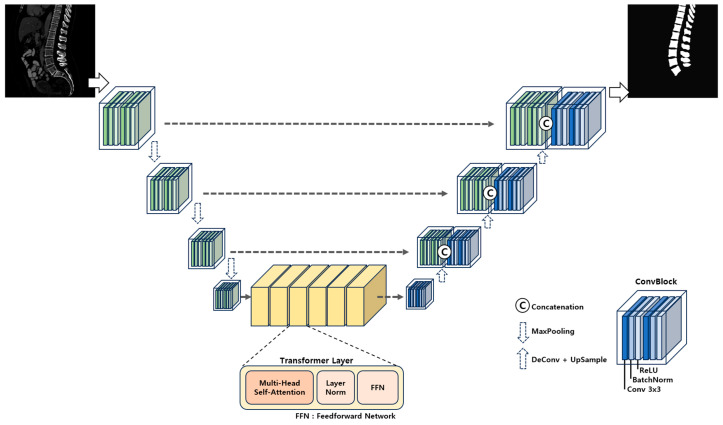
The UNETR architecture for our spine segmentation model. Building upon U-Net, we incorporated a transformer layer to extract complex spatial patterns.

Our UNETR-based segmentation model comprised two sequential convolutional layers, accompanied by batch normalization and ReLU activation functions (Figure 2). The transformer encoder employed positional encoding and multi-head attention mechanisms to capture essential information from the input feature maps, with a particular focus on spatial relationships. It was particularly adept at understanding spatial relationships and global context, complementing U-Net’s strength in capturing local features, thus facilitating more comprehensive feature learning. This made the UNETR model exceptionally well-suited for learning complex patterns, a capability that proved especially valuable in managing the diverse anatomical variations of the spine.

We utilized 243 cases of CT images (61,236 slices) from the VerSe2020 dataset for training and validating our segmentation model. To simulate low bone density for data augmentation, we artificially and randomly decreased the HU values in the trabecular bone regions of the training CT images, reflecting the lower attenuation values typically seen in patients with low bone density. We also applied data augmentation, including translation, random rotation up to 15 degrees, Gaussian noise, and blurring. After applying data augmentation, we secured 183,708 slices. The images were windowed with a center of 400 and a width of 800, then normalized to a scale of 0 to 1. To optimize both pixel-level accuracy and maintain the structural integrity of the segmentation, we combined binary cross-entropy (BCE) loss and dice loss. BCE loss targeted minimizing the pixel-level prediction error, while dice loss enhanced the overall overlap between predicted and actual masks, thereby improving the overall quality and reliability of the segmentation. The UNETR model was trained using PyTorch 2.1.0 on a GTX 3090 GPU (Nvidia, Santa Clara, CA, USA). The segmentation model was trained with a batch size of 4 for 100 epochs, using the Adam optimizer with a learning rate of 1 × 10^−4^.

#### 2.2.3. VCF Detection

We employed the YOLOv9 model for VCF detection [17]. A key benefit of the YOLO model is its remarkable speed, which comes from processing the entire image in a single pass for object detection. Unlike other models that analyze different parts of an image multiple times, YOLO scans the whole image at once, predicting the locations and categories of objects simultaneously. Moreover, YOLO tends to have a lower false positive rate compared to other detection models, as it considers the entire image when determining object positions, thus minimizing the chances of incorrect detections.

The input image is segmented into a grid of size (S × S). For instance, if S = 7, the image is divided into a 7 × 7 grid. Each grid cell is tasked with determining the presence of an object within its area. For any detected objects, each grid cell predicts multiple bounding boxes, along with their associated confidence scores. These bounding boxes are defined by four key parameters: the coordinates of the bounding box’s center (x, y) relative to the grid cell, and the width and height (w, h) relative to the entire image. The confidence score indicates the likelihood that the bounding box contains an object, combined with the probability that the object belongs to the predicted class. The YOLO model employs a loss function to evaluate and compare the predicted bounding boxes and class probabilities against the ground truth. This loss function considers discrepancies in bounding box location, size, confidence levels, and class predictions, thereby refining the model’s learning process during training.

We trained the VCF detection deep learning model using data from 435 patients, randomly split in a 3:1 ratio from the total 589 VCF patients (Figure 1). During training on the sagittal CT images of these 435 patients (139,635 slices), we first excluded the front and back slices that corresponded to the external body regions, as only the slices showing the spine were needed. This resulted in the exclusion of approximately 25% of the slices, leaving 106,443 slices. After applying data augmentation techniques, including translation and random rotation up to 15 degrees, we secured a total of 319,329 slices for training.

For training our detection model, we used spine-segmented binary masks to generate the vertebra contours. Using the spine segmentation model, we created a spine mask for each vertebra. After spine segmentation, we refined the contour of the vertebral body for VCF detection model training. Based on the binary spinal mask of each vertebra, we dilated the mask using the OpenCV Python library (version 1.18.0) and subtracted the mask of the vertebral region. We then paired the bounding box coordinates with the spine contour mask for training.

#### 2.2.4. Proposed Methods

**Figure 3:**
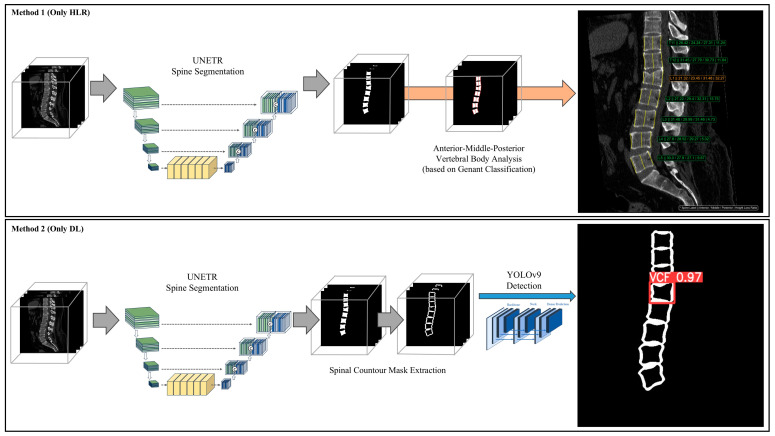

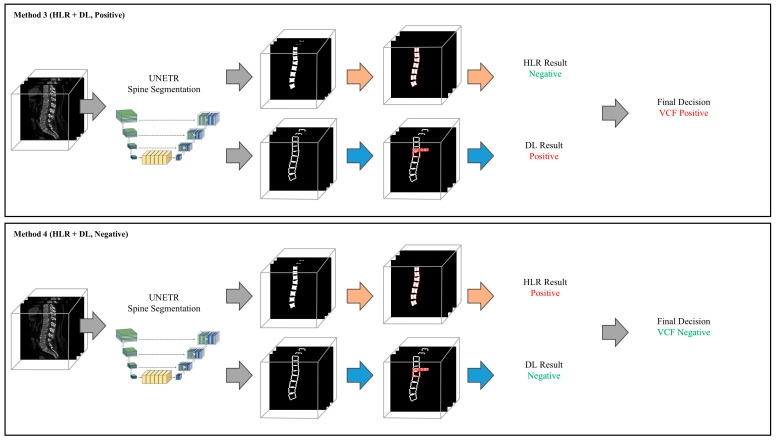
The overview of four different methods in our study. The consecutive values in the image of Method 1, simply indicated with an * symbol, were spine label, anterior height, middle height, posterior height, and height loss ratio.

In our study, we investigated the four different methods (Figure 3). First, in Method 1 (only HLR), we segmented each vertebra and measured the HLR to validate the detection performance of the HLR-based quantitative method. Second, in Method 2 (only DL), we used a spinal contour mask based on a binary vertebra mask for the VCF detection model, which can detect VCF without relying on exact measurements of vertebral height. Third, in Method 3 (HLR + DL, positive), we combined two different methods—the quantitative HLR method and the DL VCF detection model—to compensate for the errors of each method. For vertebrae classified as negative based on the Genant classification, if the detection model’s confidence score was higher than the optimal threshold, we reclassified that vertebra as positive. Lastly, in Method 4 (HLR + DL, negative), in cases where the HLR result was positive, if the detection model classified it as negative, we classified it as negative.

### 2.3. Evaluation Metrics

We assessed the accuracy of our spine segmentation model using several metrics: dice similarity coefficient (DSC), false negative dice (FND), false positive dice (FPD), intersection over union (IoU), sensitivity, specificity, F-1 score, and accuracy. These metrics were defined by specific equations, which we applied to compare the ground truth spine masks from the VerSe2020 dataset with the vertebral body masks generated by our model. The values for true positive (TP), true negative (TN), false positive (FP), and false negative (FN) were derived from the pixel-wise binary classification of the spine segmentation masks.

We also calculated the sensitivity, specificity, accuracy, and precision, measured using the confusion matrix for VCF detection performance at each vertebra. The TP, TN, FP, and FN values were based on vertebrae-wise classification results for chronic VCF detection.

The receiver operating characteristic (ROC) curve is a graphical tool used to evaluate the performance of binary classification models. This curve plots the true positive rate (sensitivity) against the false positive Rate (1-specificity) across all possible threshold values. The area under the curve (AUC) quantifies the overall ability of the model to distinguish between positive and negative classes, with values ranging from 0 to 1. We used the ROC curve to visually assess how well a model discriminates between the two classes, facilitating effective threshold selection and model comparison, while the AUC provides a single scalar value that summarizes the model’s overall predictive accuracy.

## 3. Results

The DSC of 0.944 indicated a high degree of similarity between the model’s predictions and the ground truth, reflecting the model’s strong segmentation accuracy with relatively low variability across different cases (Table 1). The FND, measured at 0.066, that very few actual vertebral regions were incorrectly classified as non-vertebrae. Also, the FPD was very low at 0.045, showing that the model rarely predicts structures that are not present, further supporting the model’s high accuracy in distinguishing between vertebral and non-vertebral regions. The IoU score of 0.894 further confirmed the model’s robust performance, indicating that the predicted segmentation overlaps significantly with the ground truth, with only a small portion of the area not aligning.

Sensitivity measures the proportion of actual positives (vertebral regions) that were correctly identified by the model. A sensitivity of 0.934 showed that the model correctly identified 93.4% of the vertebral regions. Specificity refers to the model’s ability to correctly identify non-vertebral regions. A value of 0.995 was nearly perfect, meaning that the model almost always identified non-vertebral regions correctly without misclassifying them as vertebrae. The F1 Score is the harmonic mean of precision and recall (sensitivity). A score of 0.944 indicated that the model has a strong balance between precision (how many predicted vertebral regions were correct) and recall (how many actual vertebral regions were detected). Accuracy measures the overall correctness of the model, considering both true positives and true negatives. With an accuracy of 0.989, the model was correct 98.9% of the time, which further emphasizes its robustness and reliability for spine segmentation.

**Figure 4:**
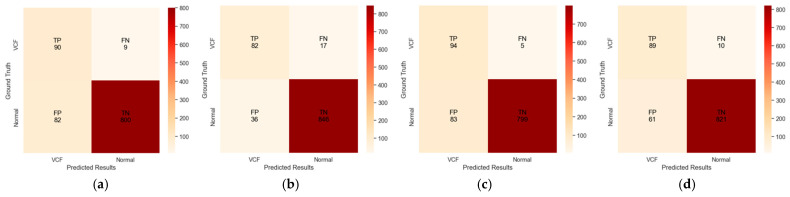
The confusion matrix for VCF detection results of four different methods. (**a**) Method 1 (Only HLR); (**b**) Method 2 (Only DL); (**c**) Method 3 (HLR + DL, Positive); (**d**) Method 4 (HLR + DL, Negative).

Using Method 1, we detected 90 true positive (TP) cases and 800 true negative (TN) cases (Figure 4). However, Method 1 incorrectly classified 82 normal cases as positive (false positives, FP) and failed to detect 9 VCF cases (false negatives, FN). Method 2 successfully identified 82 VCF cases as true positives and 846 normal cases as true negatives. It misclassified 36 normal cases as positive for VCF (FP) and missed 17 VCF cases (FN). Method 3 detected 94 true positive cases and 799 true negative cases, but it incorrectly identified 83 cases as false positives and 5 cases as false negatives. Method 4 identified 89 true positive cases and 821 true negative cases. However, it incorrectly classified 10 cases as false negatives and 61 cases as false positives.

We compared the performance of four VCF detection algorithms (Method 1, Method 2, Method 3, and Method 4) using metrics such as AUROC (area under ROC), sensitivity, specificity, accuracy, and precision (Table 2). Each method also provided detailed information with 95% confidence intervals (95% CI), allowing for an assessment of the reliability of each method’s performance.

Method 1 showed a sensitivity of 90.91% in detecting VCF, with a confidence interval ranging from 83.44% to 95.76%. The specificity was 90.70%, with a 95% CI ranging from 88.59% to 92.54%, demonstrating stable performance similar to its sensitivity. With an AUROC of 0.948, Method 1 exhibits very strong discriminatory power. The overall accuracy of 90.72% was high, reflecting a good balance between true positive and true negative predictions; however, the precision was 52.32%, meaning only about 52% of cases predicted as VCF by this method were actually true VCF cases.

Method 2 showed a sensitivity of 82.83%, which is somewhat lower than Method 1, with a CI ranging from 73.94% to 89.67%, indicating a broader range compared to Method 1. The specificity was 95.92%, higher than Method 1, indicating that this method is better at correctly classifying normal cases, with a 95% CI from 94.39% to 97.13%, which is very narrow and stable. With an AUROC of 0.923, this method performs lower than Method 1 but still shows good overall performance. The accuracy of 94.60% and precision of 69.49% were significantly higher than those of Method 1. This lower false positive rate contributed to its higher specificity and accuracy.

Method 3 had the highest sensitivity at 94.95%, with a CI ranging from 88.61% to 98.34%, showing high reliability in its sensitivity. The specificity was 90.59%, similar to that of Method 1, indicating stable performance with a CI range from 88.47% to 92.43%. With an AUROC of 0.968, this method showed the best overall performance, making it the most effective model for distinguishing between VCF and normal cases. The accuracy of 91.03% showed that this method performs well overall, though slightly lower than Method 2. However, precision was somewhat low, similar to Method 1. This indicates a higher rate of false positives, which was a trade-off for its high sensitivity.

Method 4 had a sensitivity of 89.90%, showing relatively high performance in detecting VCF. The CI ranged from 82.21% to 95.05%, indicating reliable sensitivity. The specificity was 93.08%, with a CI ranging from 91.20% to 94.67%, showing stable performance. With an AUROC of 0.947, Method 4 performs similarly to Method 1, showing strong discriminatory power. This method had a strong overall accuracy of 92.76%, showing a good balance between sensitivity and specificity. Precision was higher than Methods 1 and 3 but lower than Method 2, meaning it was fairly good at correctly identifying VCF cases when predicted.

**Figure 5:**
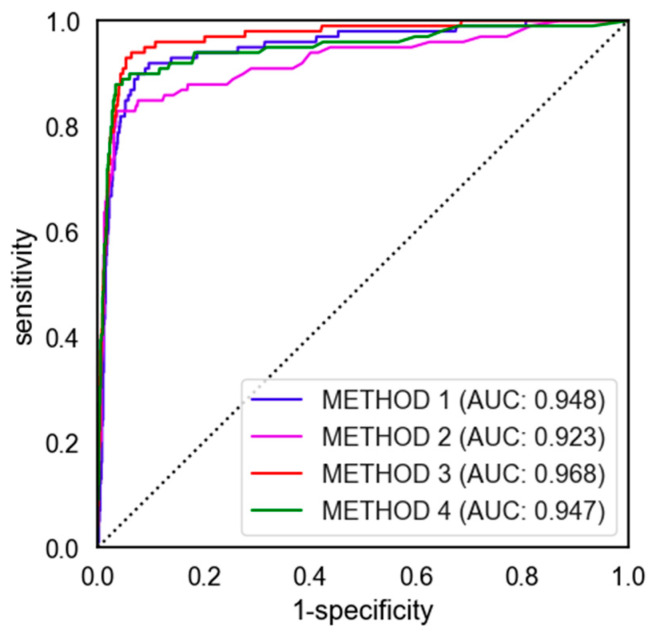
The ROC curve of four major methods.

The ROC (receiver operating characteristic) curves for the four VCF detection algorithms are illustrated in Figure 5. Each curve represents the trade-off between sensitivity and specificity for the respective method, with the corresponding area under the curve (AUC) values providing a quantitative measure of the model’s performance. The ROC curve for Method 1 is depicted in blue and shows strong discriminatory performance with an AUC of 0.948. The curve rises steeply towards the top-left corner, indicating that Method 1 effectively balances sensitivity and specificity. The high AUC suggests that this method is capable of distinguishing between VCF and normal cases with high accuracy. Method 2, represented by the purple line, has the lowest AUC of 0.923 among the four methods. The ROC curve for Method 2 is less steep compared to the other methods, particularly at lower false positive rates. This indicates that while Method 2 performs well in terms of specificity, it sacrifices some sensitivity, leading to a less optimal balance between the two metrics. The ROC curve for Method 3 is shown in red and demonstrates the best overall performance with the highest AUC of 0.968. The curve closely follows the left-hand and top borders of the graph, which is indicative of a method with excellent sensitivity and specificity. This method is particularly effective at correctly identifying VCF cases while minimizing the number of false positives, making it the most robust algorithm for VCF detection among those tested. Method 4, represented by the green line, also shows strong performance with an AUC of 0.947. The ROC curve for this method is similar to that of Method 1, with a slight trade-off between sensitivity and specificity compared to Method 3. However, the curve still demonstrates a high level of accuracy in distinguishing between VCF and normal cases, making Method 4 a viable option for VCF detection.

**Figure 6:**
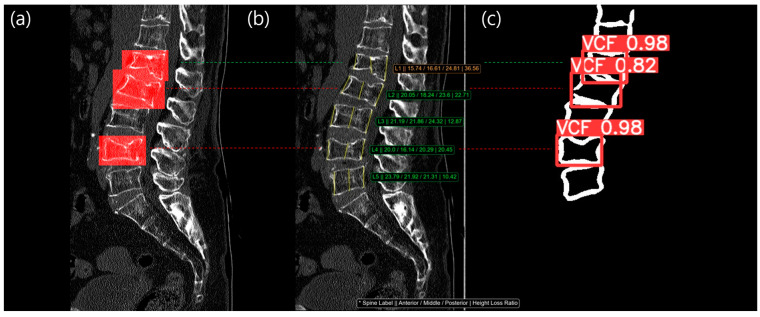
The result images of our study: (**a**) Chronic VCF reference standard labeled from radiologist; (**b**) HLR measurement, Method 1, result. The consecutive values in the image, indicated with an * symbol, were spine label, anterior height, middle height, posterior height, and height loss ratio; (**c**) VCF detection, Method 2, result from vertebral contour. At L2 and L4 level (red dotted line), HLR was normal but VCF detection model was able to locate the VCF with a high confidence score (0.82 at L2 and 0.98 at L4). The green dotted line was TP in both Method 1 (**b**) and Method 2 (**c**). The red dotted line was FN in Method 1 (**b**) but TP in Method 2 (**c**).

**Figure 7:**
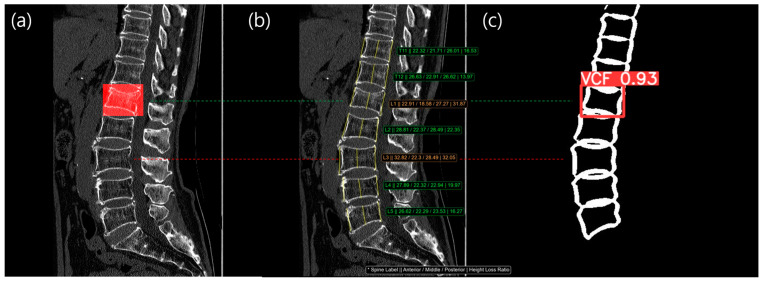
The result images from our study are as follows: (**a**) Chronic VCF reference standard labeled by a radiologist; (**b**) HLR measurement, Method 1, result. The consecutive values in the image, indicated with an * symbol, were spine label, anterior height, middle height, posterior height, and height loss ratio.; (**c**) VCF detection, Method 2, result from vertebral contour analysis. At the L3 level (indicated by the red dotted line), the HLR was 32.05%, indicating a VCF. However, the VCF detection model did not identify the VCF. The green dotted line was TP in both Method 1 (**b**) and Method 2 (**c**). The red dotted line was FP in Method 1 (**b**) but TN in Method 2 (**c**).

Figure 6 and Figure 7 showed example cases for Methods 3 and 4. The left image shows a filled red bounding box, representing the reference standard from a radiologist, overlaid on the patient’s CT images. The middle image shows the three main vertebral heights overlaid on the patient’s CT images. The right image shows the VCF detection result, with the confidence score overlaid on vertebral contour mask images. As shown in Figure 6, L2 and L4 were labeled as a VCF, but the HLR was normal, so it was not detected using method 1. However, the contour-based detection model was able to identify the VCF at L2 and L4 with a high confidence score (0.82 and 0.98, respectively), allowing Method 3 to detect the previously missed VCF case. In Figure 7, there was no red bounding box on the L3 vertebra, indicating that it was normal. However, the HLR of L3 was 32.05%, classifying it as a moderate VCF based on the Genant classification. The detection model also skipped L3, meaning Method 4 was able to identify more normal cases.

## 4. Discussion

In addition to quantitatively measuring vertebral height, incorporating morphological features is essential for accurately detecting chronic VCF. This study explored the limitations of the HLR method in identifying VCF, particularly cases clinically classified as chronic, where vertebral height alone may not suffice. We assessed the efficacy of DL in detecting morphological features of cortical bone using a vertebral contour mask. Our hypothesis was that applying a quantitative threshold based on vertebral height loss, followed by a DL-based detection model, would enhance the accuracy of VCF detection. We developed a YOLO-based detection model and combined it with the HLR method for chronic VCF detection, subsequently comparing the performance of four different approaches.

A number of methods have been proposed in the previous literature to automatically detect VCFs in CT images. John et al. evaluated the diagnostic performance of a DL-based VCF algorithm with 1087 study participants [18]. The sensitivity and specificity for moderate/severe VCF, which was defined by HLR > 25% according to the Genant classification, were 0.78 (95% CI, 0.70–0.85) and 0.87 (95% CI, 0.85–0.89), respectively. Syed et al. proposed vertebral deformity fracture detection using vertebral height features and parametric computational modeling [19]. Contour analysis was performed on the central anterior-posterior plane, and the anterior-to-posterior ratio and middle-to-posterior ratio metrics were calculated to quantify fracture deformities. In the test dataset, expert readers identified biconcave and wedge VCFs with moderate or severe (HLR > 25%) deformities on T1-L1 vertebrae. The sensitivity and specificity were 94.8% and 98.5%, respectively, for 40,050 vertebrae from 3231 chronic obstructive pulmonary disease patients. Joseph et al. used vertebral height distribution for analysis [20]. The height distribution across the vertebral body was computed by partitioning the axial cross-section of a vertebral body into 17 sectors. The pattern of the height was analyzed using support vector regression to differentiate between fractured and normal vertebrae. This method achieved a sensitivity of 95.7% and a false positive rate of 0.29 from a total of 1275 vertebrae in 150 CT examinations. These studies only used vertebral height to diagnose VCF and did not consider chronic VCF classification.

In our study, we discovered that when we used only the HLR method, we could not ensure accurate detection of chronic VCFs, which were clinically labeled as ground truth by radiologists. In real clinical practice, height loss is not the sole criterion for diagnosing chronic VCF. Although some studies, such as that by Syed et al. [19], demonstrated state-of-the-art performance in detecting moderate/severe VCF, it is essential to consider the performance in chronic VCF as well.

Likewise, there were some studies using commercial software for detecting VCF. Magnus et al. evaluated the performance of automatic VCF detection software (HealthVCF, Version 5.1.1), which used an HLR-based algorithm, in a real-life setting at a Danish hospital [21]. The evaluation of 1000 CT scans revealed a sensitivity of 0.68 and a specificity of 0.91. They demonstrated that HealthVCF, which focused solely on the HLR method, performed worse than expected, and the tested version was not generalizable to the Danish population. A similar study by Renata et al. reported that the HealthVCF software showed a diagnostic accuracy of 89.6%, with a sensitivity of 73.8% and a specificity of 92.7% from 899 chest and abdominal CT scans [22]. It showed that the HLR-based algorithm could achieve high specificity but low sensitivity. Therefore, as in our study, integrating deep learning can enhance detection performance.

Recently, some studies have focused on using DL methods to detect VCF without relying on HLR. Sankaran et al. proposed a bounding box-based CNN classification method for the automatic detection of VCFs [23]. They generated six different 3D bounding boxes and split them into 2D sagittal slices around the coronal center. Each slice was divided into patches for training the CNN classification to detect VCF, followed by majority voting. The average three-fold cross-validation showed a sensitivity of 88.10% and a specificity of 84.20% on 308 chest CT patients. Wongthawat et al. evaluated the detection potential of DL for osteoporotic VCF [24]. They employed the YOLOv8 architecture for high-performance object detection, and the validation data was classified using the AO Spine-DGOU (German Society for Orthopaedics and Trauma) Osteoporotic Fracture Classification System (OF 1 to OF 5) [25]. This study divided the 1050 sagittal CT-scan radiographic images of the thoracolumbar region into 934 cases for the training dataset and 116 cases for the test dataset. The sensitivity ranged from 95.85% to 97.35%, and the specificity ranged from 94.28% to 97.86% across grades OF 1 to OF 4.

We expanded on the concepts of previous studies by conducting this research to examine the effectiveness of combining both HLR and deep learning methods, rather than relying solely on either HLR or DL to detect chronic VCF. Method 3 (HLR + DL, positive) demonstrated the best overall performance for VCF detection, achieving the highest AUC of 0.968. This indicates that Method 3 provides the optimal balance between sensitivity and specificity, making it the most effective model for accurately identifying VCF cases while minimizing false positives. The high AUC reflects its ability to correctly classify a large majority of both positive and negative cases, ensuring that the model is both reliable and robust across different threshold levels. Method 2 (only DL) stands out for its exceptional specificity, meaning it is particularly adept at reducing false positive rates. However, this increased specificity comes at the cost of sensitivity, resulting in a model that, while strong in avoiding false alarms, may miss a greater number of true VCF cases. The lower AUC of 0.923, compared to the other methods, suggests that Method 2 is less effective overall due to this trade-off, especially in scenarios where detecting all possible cases is crucial. Both Method 1 (only HLR) and Method 4 (HLR + DL, negative) offer well-balanced performances with high AUCs of 0.948 and 0.947, respectively. These methods strike a solid balance between sensitivity and specificity, making them dependable alternatives depending on the specific requirements of the detection task. Whether the priority is to maintain a balanced detection rate or to slightly favor minimizing false positives, both methods provide reliable results, ensuring effective VCF detection across various clinical settings.

From Figure 6 and Figure 7, we identified two common limitations of the HLR (height loss ratio) method. First, when only the height loss ratio was applied, it became difficult to detect vertebral bodies that were uniformly deformed across the anterior, middle, and posterior regions using the HLR method. In some cases, even though the deformity of the cortical bone was clearly visible, the vertebral height remains uniform, making it challenging to detect VCF using the HLR method. In such instances, we found that utilizing the deformity of the cortical bone was a more effective approach for identifying VCF. Second, although there may appear to be a significant height loss, the calculated height loss ratio may still fall below the threshold (25%) required for diagnosing VCF. This issue arose from the fact that the height loss ratio varies depending on the size of the vertebral body, even if the absolute height loss was the same. For example, suppose both vertebral body A and vertebral body B experience a 5 mm height loss. In the case of vertebral body A, if it originally measured 15 mm, the height loss ratio was calculated as 33% (1 − 10/15), which qualifies for a VCF diagnosis. However, for vertebral body B, if the original height was 25 mm, the height loss ratio was calculated as 20% (1 − 20/25), which does not meet the criteria for a VCF diagnosis. This was the critical limitation of HLR method.

The deep learning method also has its limitations. In the DL method, the number of false negatives, i.e., where VCF is present but the model failed to detect it, increased. Additionally, the number of false negatives where there was no VCF (correctly predicted as negative) also significantly increased. This phenomenon was likely due to the imbalanced nature of the training data. In this study, the data used for training reflected the real-world prevalence of VCF in clinical settings, meaning that the majority of vertebrae in the training data were normal (negative), which led the deep learning model to become better at identifying normal cases. To address this data imbalance during training, we augmented the limited number of positive VCF vertebrae images and used them in the training process. However, the absolute number of positive VCF cases used in the study was still insufficient. Future studies should include a larger number of positive VCF cases to improve the training process and address this issue.

Our study has several limitations. First, the sample size was relatively small, as it was a single-center study. However, the process of labeling clinical VCFs was time-consuming, making it challenging to obtain a larger, fully labeled dataset. Despite the small dataset, it was meaningful to investigate clinically classified VCFs. Second, our study focused exclusively on chronic fractures, rather than acute VCFs. Acute VCFs are often more critical in clinical settings, and our results may not fully reflect the performance of detection models in identifying acute cases. In subsequent studies, we will investigate the feasibility of applying our proposed model to acute VCFs. Lastly, our evaluation was conducted on a vertebra-wise basis rather than a patient-wise basis. While this approach does not provide a comprehensive assessment of the model’s performance at the patient level, it allowed for the detailed analysis of each vertebra. Further studies with larger, multi-center datasets are needed to validate our findings and to explore the model’s performance in detecting acute VCFs. Additionally, expanding the evaluation to include patient-level analysis would provide a more holistic understanding of the model’s clinical utility.

## 5. Conclusions

In conclusion, this study revealed the limitations of the HLR method in identifying clinically classified VCF vertebrae. We introduced a novel approach for detecting VCF features using a vertebral contour-based detection model. By combining the quantitative HLR method with qualitative deep learning techniques, we demonstrated an improved detection performance for chronic VCFs. This integrated algorithm underscores the potential for enhanced diagnostic accuracy in clinical settings.

## Figures and Tables

**Table 1:** Spine segmentation results.

DSC	FND	FPD	IoU	Sensitivity	Specificity	F-1 Score	Accuracy
0.944	0.066	0.045	0.894	0.934	0.995	0.944	0.989

**Table 2:** VCF detection performance of four different methods.

	AUROC	Sensitivity	Specificity	Accuracy	Precision
Method 1 (Only HLR)	0.948	90.91% (83.44–95.76%, 95% CI)	90.70% (88.59–92.54%, 95% CI)	90.72% (88.75–92.38%, 95% CI)	52.32% (44.90–59.66%, 95% CI)
Method 2 (Only DL)	0.923	82.83% (73.94–89.67%, 95% CI)	95.92% (94.39–97.13%, 95%CI)	94.60% (93.00–95.85%, 95% CI)	69.49% (60.68–77.08%, 95% CI)
Method 3 (HLR + DL, Positive)	0.968	94.95% (88.61–98.34%, 95% CI)	90.59% (88.47–92.43%, 95% CI)	91.03% (89.08–92.66%, 95% CI)	53.11% (45.77–60.31%, 95% CI)
Method 4 (HLR + DL, Negative)	0.947	89.90% (82.21–95.05%, 95% CI)	93.08% (91.20–94.67%, 95% CI)	92.76% (90.97–94.22%, 95% CI)	59.33% (51.33–66.87%, 95% CI)

## Data Availability

The datasets generated or analyzed during the study are available from the corresponding author upon reasonable request.
